# Hyperbaric Oxygen Therapy for Venous Leg Ulcers: A 6 Year Retrospective Study of Results of a Single Center

**DOI:** 10.3389/fmed.2021.671678

**Published:** 2021-07-28

**Authors:** Rutger C. Lalieu, Ida Akkerman, Rob A. van Hulst

**Affiliations:** ^1^Hyperbaar Geneeskundig Centrum, Rijswijk, Netherlands; ^2^Department of Anesthesiology, Amsterdam University Medical Center, Amsterdam, Netherlands; ^3^Independent Researcher, De Nieuwe Delta, Ede, Netherlands; ^4^Department of Surgery, Amsterdam University Medical Center, Amsterdam, Netherlands; ^5^Hyperbaric Department, Amsterdam University Medical Center, Amsterdam, Netherlands

**Keywords:** retrospective study, venous leg ulcer, wound healing, hyperbaric oxygen therapy, HBOT 2

## Abstract

**Background:** Venous leg ulcers (VLUs) are common and have a large impact on healthcare budgets worldwide. Hyperbaric oxygen therapy (HBOT) may improve healing of these ulcers.

**Methods:** Retrospective, single-center cohort study between 2013 and 2019. All patients with a VLU from an outpatient clinic providing HBOT and wound care were included. The primary outcome measure was wound healing, determined at discharge from the center. Other outcome measures were improvement in patient related outcome measures (PROMs), as assessed by the EQ-5D-3L questionnaire and including quality of life (QoL) and pain score.

**Results:** Fifty patients were included, 53% female, with a mean age of 73.4 (±12.2). Most wounds (83%) had existed longer than 3 months before starting treatment. Patients received an average of 43 (±20) sessions of HBOT. After treatment, 37 patients (63%) achieved complete or near-complete wound healing. Wound size decreased from a median of 14 cm^2^ [interquartile range (IQR) 32 cm^2^] to 0.5 cm^2^ (IQR 5.3 cm^2^), a median decrease of 7.5 (IQR 16.2 cm^2^) in cm^2^ (94%). Patients mostly reported improvement for all health aspects on the questionnaire. Pain score decreased from 5.7 (±2.5) to 2.1 (±2.2) (*p* < 0.0001) and health score increased from 57.2 (±15.6) to 69.9 (±18.9) (*p* = 0.02).

**Conclusions:** Patients with non-healing VLUs may benefit from HBOT to achieve complete or substantial wound healing. We recommend a well-designed randomized clinical trial with a number of patients allowing enough statistical power, and of a reasonable duration, to establish the potential of additional HBOT on hard-to-heal venous ulcers.

## Introduction

Venous leg ulcers are skin defects of the lower leg which are caused by chronic venous insufficiency (CVI). In the United States, CVI affects up to 35% of the adult population and around 4% suffers from a leg ulcer (1). For all lower extremity ulcers, CVI is most common etiology (2). Venous ulcers are more prevalent among female and elderly patients (3), reduce health-related quality of life (1, 2) and represent a major economic burden, with about 1% of healthcare budgets for most industrialized countries being spent on this disease (4). Due to the improved life expectancy, these numbers are expected to increase over the coming years.

The underlying pathophysiology is based on venous hypertension, which can be caused by deep vein thrombosis, ambulatory venous hypertension, insufficiency in the venous system itself or decreased pump action of the calf muscle (5, 6). There are surgical treatment options to correct vascular disorders (7), but the gold standard in treatment remains compressive bandaging, which aims to counteract the increased hydrostatic pressure in CVI and thereby increase venous return (8, 9). But even with this evidence-based treatment, 33 to 60% of ulcers remain unhealed after 6 weeks, (5) and 15–30% persist after a year of compression therapy (10).

There are two prevailing theories on the chronicity of venous leg ulcers (VLU). The *fibrin cuff theory* (11) states that fibrin gets deposited around capillary beds due to the increased hydrostatic pressure, which in itself further increases intravascular pressure. The fibrin deposits decrease oxygen permeability and cause local tissue hypoxia, impairing wound healing. The *trap hypothesis* (12) builds upon the fibrin cuff theory and posits that endogenous growth factors and inflammatory cells are trapped in the fibrin cuff. Besides local tissue hypoxia, this leads to a pro-inflammatory state and dysregulation of certain cytokines (5), creating a unfavorable environment in which wound healing is debilitated.

Improving tissue oxygenation and thereby wound healing is the main rationale for hyperbaric oxygen therapy (HBOT). This is achieved by placing individuals in a pressure chamber, where they breath 100% oxygen under increased atmospheric pressure. This hyperoxic state leads to an increased production of reactive oxygen species (ROS) and reactive nitrogen species (RNS), which act as signaling molecules for several cascades and pathways for growth factors, cytokines and hormones (13). This leads to, among other effects, increased angiogenesis, modulation of inflammatory activity, improved collagen deposition, and reduction of edema (13–15). The Undersea and Hyperbaric Medical Society (UHMS) has defined several indications for the therapy, including delayed radiation injury, compromised skin grafts and diabetic foot lesions (16). The therapy is considered cost-effective when treating diabetic foot ulcers (17, 18) and is safe (19), with few and usually mild side-effects. (20).

As of yet, VLU is not a recognized indication for HBOT. One reason for this is the paucity of evidence on the effect of HBOT on VLU healing, with only two small randomized clinical trials (RCTs) having been performed in the past decades. In 1994, Hammarlund and Sundberg (21) found a significant reduction in wound surface area after six weeks of HBOT, compared to standard treatment (i.e., compression stockings) combined with hyperbaric air. More recently, in 2018, Thistlewaite et al. (22) came to a similar conclusion after 12 weeks of either HBOT or placebo treatment.

In our experience, non-healing venous ulcers may benefit from additional treatment with HBOT when regular treatment options are not sufficient. Therefore, we performed a retrospective cohort study of all patients treated in our center with a venous ulcer, with the hypothesis that the addition of HBOT to standard treatment leads to wound healing for ulcers that have not respond to standard care only.

## Materials and Methods

The study design is a single center retrospective cohort study without a control group, according to the STROBE guidelines. All participants gave written informed consent at the start of the therapy and all principles of the Declaration of Helsinki were followed. The methods for handling personal details and privacy are in accordance with national and European legislation, as well as the scientific integrity guidelines of the Association of Universities in the Netherlands (23).

Patients with a VLU are referred to our center to precondition the wound for skin grafting; all these patients were included in the current study. Diagnosis and any previous treatments were performed in the referring hospital; unfortunately, no detailed classification was available for venous insufficiency in the current database. Duration of the wound is categorized as 1 (0–3 weeks), 2 (3–6 weeks), 3 (6 weeks−3 months), 4 (3–18 months) and 5 (>18 months). When patients were entered more than once in the database, only one entry was randomly selected for inclusion. In addition to HBOT, patients received surgical wound care once per week from a vascular surgeon and wound care nurse, according to local best practice (i.e., compressive bandaging and weekly surgical debridement, antibiotic therapy, and wound treatment materials on indication). Wounds were measured manually and photographed at each visit. Quality of life (QoL) questionnaires are provided to be filled out before and after therapy by all patients who are treated at our center.

### Hyperbaric Treatment

HBOT sessions take place 5 days per week (excluding the weekend), lasting 110 min per session in total. During the first 10 min of a session, the treatment chamber is pressurized to 2.4 atmospheres absolute (ATA; 240 kPa). Under this increased atmospheric pressure, patients breathe 100% oxygen for three times 20 min, with 5-min air breaks in between. The fourth and final block lasts 15 min, after which decompression is started. During decompression, patients still breathe oxygen for 8 min. In the last 2 min of decompression, patients breathe air.

The standard treatment protocol is 10–30 HBOT sessions to precondition the wound for skin grafting, depending on the formation of granulation tissue. After the procedure, 10 more HBOT sessions are done to improve take and healing of the graft. If the wound would achieve (near-)complete healing before grafting can take place, the procedure is canceled.

### Outcome Measures

The primary outcome measure was wound healing, determined at discharge from the center. The results are stratified according to a self-composed outcome classification. Closed wounds were categorized as 1. Wounds were classified as category 2 (near-complete healing) based on the research of Wicke et al. (24): wound surface healing percentage >80%, depth of the wound was at most 0.5 cm, there was 100% tissue granulation, epithelization of all wound borders and no clinical signs of infection. Besides category 1 (complete healing), category 2 is also deemed a positive outcome, since robust healing during treatment is a good predictor of complete healing (24, 25). Category 3 means no significant wound healing and category 4 includes all deteriorated wounds.

Other outcome measures were improvement in patient related outcome measures (PROMs), as assessed by the EQ-5D-3L questionnaire (www.euroqol.org). The EQ-5D questionnaire assesses mobility, self-care, daily activities, pain/discomfort, and anxiety/depression. Patients indicate whether they have no, moderate, or severe complaints in any of these domains. The questionnaire also lets people score self-perceived QoL on a hundred-point scale and pain on a ten-point visual analog scale (VAS).

### Statistical Analyses

Descriptive statistics for continuous variables were given as mean with standard deviation in case variables were normally distributed, or as medians with inter quartile ranges if not. Discrete variables were given as numbers and percentages.

The change in EQ5D domain scores before and after treatment were tested with the Wilcoxon signed rank test. The change in pain or health scores before and after treatment were tested with a *t*-test (**Figure 2**). Because surface measures were not normally distributed, change in wound surface was tested with a Wilcoxon signed-rank test. The association between curation and categorized variables (crude OR) were determined by a Pearson's chi-square test.

Logistic regression was used to relate curation (defined as wound curing categories 1 and 2) to explanatory variables. The selection of covariables for the final model was as follows. All potential covariables were added to model. Gender and age were standard variables in the model. Each other covariable was tested separately. It remained in the model if the outcome measure (odds ratio) would change more than 5% when the variable was excluded from the full model. The *p*-value of log likelihood test is presented.

A linear regression was used to investigate the relationship between the (log transformed) change in wound surface and explanatory variables. The selection of covariables for the final model was as follows. All potential covariables were added to model. Gender and age were standard variables in the model. Each other covariable was tested separately. It remained in the model if the other coefficient would change more than 10% when the variable was excluded from the full model. The *p*-value of the *F*-test is presented.

The mean healing time expressed as number of sessions or number of days, was determined with the summarizing of survival time data of STATA.

Statistical analyses were performed using the STATA version IC/16.1 software package.

## Results

A total of 59 patients was included. General characteristics of patients are described in Table 1, such as sex, age, ulcer size, and duration of the wound. The majority (53%) is female with a mean age of 73.4 (± 12.2) years. The majority of wounds (83%) had existed for more than 3 months before starting treatment.

**Table 1:** General description of the population.

		**Total**	**Healed**	**Not healed**
*N*		59	37	22
Male sex	*N* (%)	28 (47)	18 (48.6)	10 (45)
Age (years)	Mean (sd)	73.7 (12.2)	73.3 (12.1)	73.8 (12.6)
Curation (%)	Mean (sd)	73.2 (33.1)	94.0 (7.7)	36.6 (28.3)
Number of sessions (N)	Mean (sd)	43.4 (20.2)	47.2 (20.2)	37.0 (18.9)
Graft	*N* (%)	14 (23.7)	10 (27)	4 (18)
Wound duration	*N* (%)			
2 (3–6 weeks)		3 (5.1)	2 (5.4)	1 (4.5)
3 (6 weeks−3 months)		7 (11.9)	5 (13.5)	2 (9.1)
4 (3–18 months)		27 (45.8)	20 (54.0)	7 (31.8)
5 (>18 months)		22 (37.3)	10 (27.0)	12 (54.6)
Size wound start (cm^2^)	Median (IQR)	14 (32)	14 (35)	9 (16)
Size wound end (cm^2^)	Median (IQR)	0.54 (5.3)	0.04 (0.54)	5.2 (15.2)
Δ wound size (cm^2^)	Median (IQR)	−7.5 (16.2)	−13.9 (31.2)	−0.72 (6.0)
Δ wound size (%)	Median (IQR)	−94 (55)	−100 (3.8)	−13.7 (67.6)
Pain score start (1–10)	Mean (sd)	5.7 (2.5)	5.8 (2.6)	5.5 (2.4)
Pain score end (1–10)	Mean (sd)	2.1 (2.2)	1.5 (1.9)	3.6 (2.5)
Δ pain score^*^	Mean (sd)	−3.5 (2.4)	−3.8 (2.0)	−2.8 (3.2)
Health score start (0–100)	Mean (sd)	57.2 (15.6)	56.2 (15.8)	58.8 (15.7)
Health score end (0–100)	Mean (sd)	69.9 (18.9)	69.7 (19.6)	70.5 (17.9)
Δ health score^*^	Mean (sd)	8.6 (23.6)	10.4 (25.5)	4.3 (18.8)

*sd, standard deviation; IQR, interquartile range*.

* *The number of participants that filled in health questionnaire both at start and end was 34*.

Patients received an average of 43 (±20) sessions of HBOT. Most patients (78.9%) received 30 sessions or more. After treatment, 37 patients (63%) achieved complete or near-complete wound healing, with an average of 47.2 HBOT sessions used. The remaining 22 patients (37.3%) did not achieve clinically significant wound healing.

**Figure 1:**
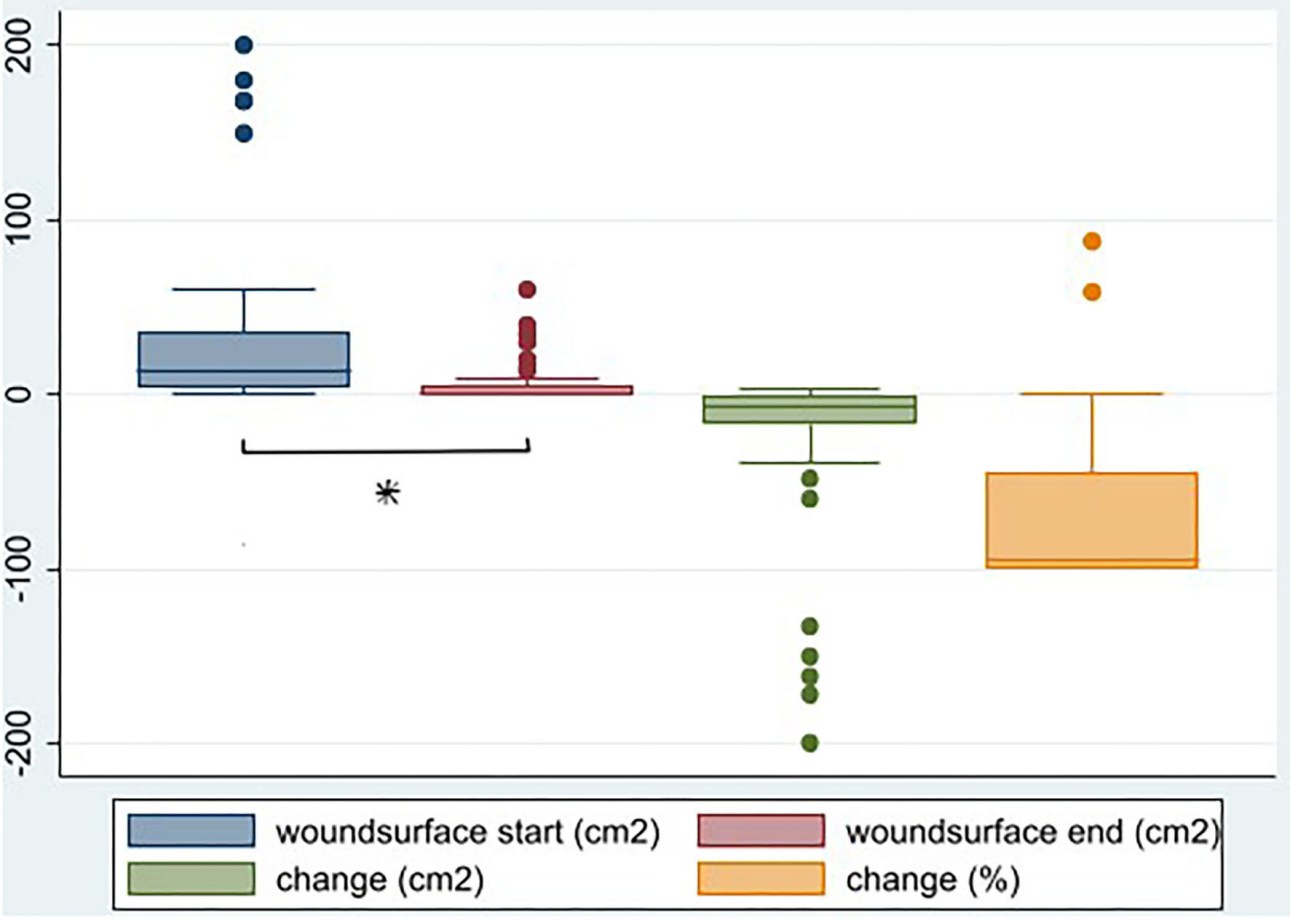
Wound surface before and after HBOT (cm^2^), change in wound surface (surface after—surface before in cm^2^) (*p* < 0.001), and as percentage of surface before (%), *N* = 59. *Denotes statistical significance.

Wound surface area was significantly lower after treatment (*p* < 0.001). The median wound size decreases from 14 to 0.5 cm^2^, a median decrease of 7.5 in cm^2^ (94%). Noteworthy is that two patients show a relatively big increase in wound surface area, though small in absolute measures (Table 1 and Figure 1).

Curation, defined above as category 1 and 2, is significantly associated with wound duration at start, as well as number of sessions (Table 2).

**Table 2:** Association between curation and explanatory factors.

	**Univariate**				**Multivariate**		
	**CrudeOR**	**95% C.I**.	***p*-value**		**OR**	**95% C.I**.	***p*-value**
Sex (male = 1)	1.14	(0.39–3.31)	0.814	Sex (male = 1)	0.53	(0.15–1.93)	0.336
Age (>73.7 = 1)	0.98	(0.34–2.85)	0.971	Age (y)	0.99	(0.94–1.04)	0.587
Wound duration (cat 5 = 1)	0.31	(0.09–0.99)	0.036	Wound duration (1–5)	0.42	(0.18–0.99)	0.048
Number of sessions (≥44 = 1)	1.70	(0.58–5.03)	0.333	Nr of sessions (nr)	1.04	(1.00–1.08)	0.026
Graft (1 = yes)	1.67	(0.4 4–6.25)	0.444				

*Results of univariate calculation and of multivariate logistic regression (graft did not remain in final model), N = 59*.

The decrease in wound surface area (in cm^2^) as a continuous variable can be related by linear regression [after log transformation; LTsurface = ln(–Δ wound size + 4)] to explanatory variables (Table 3). Number of sessions is significantly associated with wound surface area, statistically as well as clinically, while duration of the wound is not. The median healing time in this population is 54 sessions, or 120 days.

**Table 3:** Association of decrease in wound surface area with explanatory variables.

	**Univariate**			**Multivariate**		
**LTsurface**	**Coeffi-cient**	**95% C.I**.	***P-*value**	**Coeffi-cient**	**95% C.I**.	***P*-value**
Sex (male = 1)	−0.03	(−0.68 to 0.62)	0.933	−0.28	(−0.87 to 0.30)	0.334
Age	0.003	(−0.03 to 0.03)	0.847	−0.01	(−0.03 to 0.02)	0.724
Wound duration	−0.04	(−0.43 to 0.36)	0.852	−0.20	(−0.56 to 0.16)	0.280
No. of sessions	0.032	(0.02 to 0.05)	0.000	0.04	(0.02 to 0.05)	0.000
Graft	0.309	(−0.45 to 1.06)	0.415			
Constant				2.61	(−0.09 to 5.31)	0.060

*Results of the linear regression after log transformation, N = 53*.

**Figure 2:**
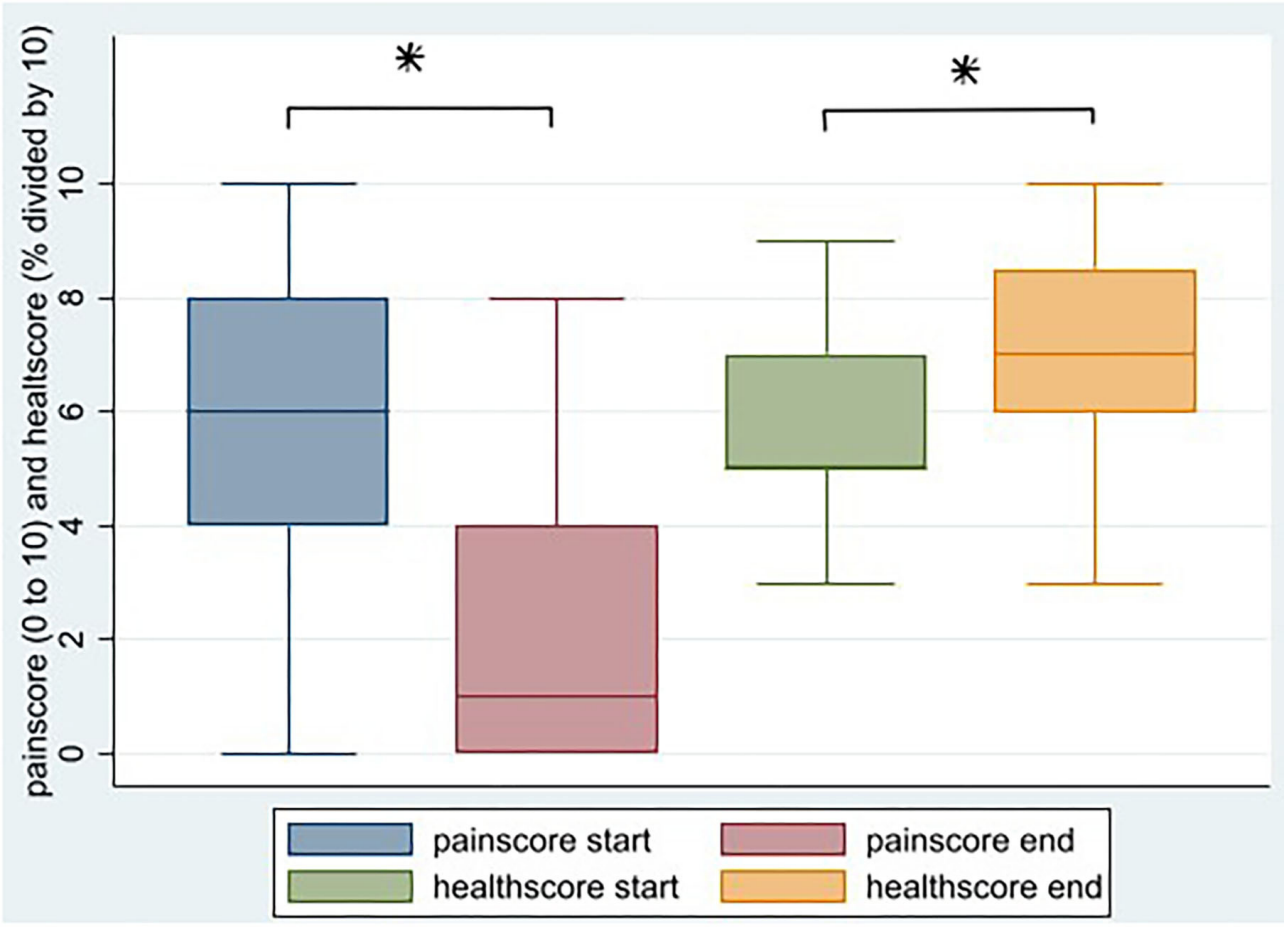
Change in self-reported pain score and self-reported health score after HBOT, *N* = 34 (*p < 0.05).

Thirty-seven patients (62%) filled in the EQ5 health questionnaire before and after treatment. Most patients reported improvement on all domains, and the number of patients reporting improvement was statistically significant for mobility and for pain/discomfort. Likewise, the self-reported pain score decreases from 5.7 to 2.1 (*p* < 0.001) and the self- reported health score increases from 57.2 to 69.9 (*p* = 0.02; Table 1 and Figure 2).

## Discussion

This retrospective cohort study shows that patients with chronic VLUs may benefit from adjunctive HBOT to achieve complete or substantial wound healing. Most of the patients achieved complete healing (31%), or near-complete healing (34%). Overall, there was a median of 94% reduction of wound surface area after treatment. Furthermore, patients reported an increased health score, and a lower pain score.

Because most of the patients, even those who did not acquire complete healing, seemed to profit from the addition of HBOT, we have tried to identify the factors that influenced the process of wound healing in this population, to determine possible selection criteria.

Factors of influence appeared to be number of sessions and duration of the wound before start. Age is a known limiting factor in wound healing (26), but in this population its influence was apparently diminished, possibly due to the effect of HBOT.

The number of sessions was positively associated with curation (Table 2): the higher the number of sessions, the larger the decrease in wound size (*p* = 0.020; Table 3). Wound duration before start was also associated with curation (Table 2). The statistically significant odds ratio of 0.42 can be interpreted as that a wound that has healed, has 58% higher odds of having been of one duration category lower than a patient that not healed. Clinically, the more severe wounds often are the larger ones, and so the higher the duration category, the longer the healing time. There was, however, no statistically significant association of wound duration with wound size reduction (Table 3). Regression analysis with the percentual size reductions did not reveal an association with wound duration. However, these results were not statistically significant, probably due to the low number of patients in the lower categories, impeding statistical power.

Factors that did not influence wound healing are gender and the application of graft surgery. While most patients were referred for HBOT as preparation for a skin graft to close the wound, only 15 patients (25%) ultimately underwent this procedure. Of these patients, five (33%) achieved complete healing and six (40%) reached a mean of 90% healing. The four remaining patients (27%) did not achieve substantial healing, but still recorded a mean of 55% reduction. Of the 44 patients who did not receive a graft, 14 (32%) still achieved complete healing and another 14 (32%) near-complete healing, with a mean wound surface reduction of 88%. It is possible that the more severe cases required grafting, counteracting a statistical association with healing, but this remains speculative.

As a practical application of these factors, the coefficient of 0.04 in the linear regression (with log transformation) would indicate that a man of 70 with wound duration category 3 (6 weeks−3 months), having had 10 HBOT sessions, would on average achieve another 2.1 cm^2^ wound size reduction when undergoing another 10 sessions.

We have compared our results to available literature on HBOT for venous ulcers. We identified only one other cohort study, from 1970, in which 17 out of 19 VLU patients (89%) treated with HBOT were healed (27).

The number of RCTs comparing HBOT to standard wound care is limited. The study of Hammarlund and Sundberg (21) is cited several times (28, 29) in this context, but the control group did receive air in a pressurized chamber. The expected effect of this hyperbaric air on wound healing is small. Indeed, no significant wound healing is noted in the control group. Interestingly, on the basis of this trial, Angle and Bergan (30) stated that “…it [HBOT] should not be considered as treatment”; a conclusion that cannot be justified since the HBOT group did ultimately achieve significant wound healing compared to the control group.

Thistlethwaite et al. (22) conducted a trial with 31 patients with VLUs failing to heal after 4 weeks of standard treatment. The median duration of the wound was 48 weeks. The number of patients did not meet the 58 as estimated by the authors for sufficient power. Combined with the limited follow up time of 12 weeks, this might have caused the absence of a significant difference in the numbers of completely healed wounds between the groups. The HBOT intervention group had a mean of 95% area reduction (PAR), compared to a mean 54 PAR for the placebo group. The PAR in the treatment group is comparable to our findings of 94%.

Longobardi et al. (31) compared two different regimes of HBOT, namely twice a day for 3 weeks or once daily for 6 weeks, with standard treatment in a group of 81 patients with hard to heal venous ulcers. Healing was defined as a minimum wound area reduction of 40% and was highest in the 6-week HBOT group, and lowest in the 3-week HBOT group. Wound surface reduction occurred in all groups but was not significantly higher in HBOT groups; even significantly lower in the 3-week group compared to standard treatment. The authors conclude that the addition of HBOT can lead to increased wound healing, when applied for a longer, but less intensive treatment period.

Since a significant portion of VLUs remain unhealed after conventional therapies, (5, 10) it is necessary to identify and explore additional treatment options. The results of the current study all confirm the results of these earlier studies: HBOT combined with conventional wound care may improve wound healing of otherwise non-healing VLUs.

There are some limitations to the current study. First, since this is a retrospective cohort study, there is an inherent risk of confounding. Factors that are known to influence wound healing, such as smoking and diabetes (32), were not recorded in the current database. The diagnosis of venous insufficiency was made in the referring hospital, and detailed information on classification (e.g., CEAP) was not provided. This information would have strengthened the analyses of the results, and provided information on generalisability of the population. However, it would not have altered the general conclusion that VLUs may benefit from HBOT to improve their overall healing capacity. Secondly, we used a generous definition of curation, but we were confident that nearly closed wounds would heal in little time, as found in earlier research (24, 25). Thirdly, we do not know how wound healing would have progressed without HBOT, since there is no control group. However, the fact that most wounds existed for more than 3 months when HBOT started, indicates that the possibility of spontaneous healing was low. Ideally, a (blinded) randomized trial is necessary to determine the effect of the addition of HBOT to standard treatment more accurately. Unfortunately, at this point there is no consensus on the sham HBOT that a control group should undergo, which makes RCTs more difficult to design (33).

Finally, the follow-up period is limited. After patients finish their treatment in our center, they are referred back to their treating physician for further follow-up and treatment. While we do send out questionnaires after 3, 12, and 24 months, these are seldom returned. Therefore, we have no clear data on relapse of the wounds. We would argue that a relapsing is not necessarily a failure of HBOT, since this a curative treatment and not preventative. Adequate care for venous insufficiency (e.g., compressive bandaging) should be continued to prevent new or relapsing ulceration.

Conversely, a strength of the study is that our database is of a considerable size not stated in literature yet, and we have had the opportunity to follow the patients for several months. In spite of the severity of the wounds, we do find promising results with HBOT in this otherwise difficult-to-heal population.

In conclusion, HBOT may be employed to improve wound healing in patients with a venous leg ulcer, who have not responded to standard wound care. This increased wound healing may be utilized to prepare the wound bed for skin grafting, or to achieve wound healing without a graft. We recommend a well-designed randomized clinical trial with a number of patients allowing enough statistical power, and with a reasonable follow-up duration, to establish the potential of adding HBOT to standard wound care for hard-to-heal venous ulcers.

## Data Availability Statement

The data analyzed in this study is subject to the following licenses/restrictions: the database will not be publicly available. Requests to access these datasets should be directed to r.lalieu@hgcrijswijk.nl.

## Ethics Statement

Ethical review and approval was not required for the study on human participants in accordance with the local legislation and institutional requirements. Written informed consent for participation was not required for this study in accordance with the national legislation and the institutional requirements.

## Author Contributions

RL and RH contributed to conception and design of the study. RL organized the database. IA performed the statistical analysis. RL wrote the first draft of the manuscript. All authors contributed to manuscript revision, read, and approved the submitted version.

## Conflict of Interest

The authors declare that the research was conducted in the absence of any commercial or financial relationships that could be construed as a potential conflict of interest.

## Publisher's Note

All claims expressed in this article are solely those of the authors and do not necessarily represent those of their affiliated organizations, or those of the publisher, the editors and the reviewers. Any product that may be evaluated in this article, or claim that may be made by its manufacturer, is not guaranteed or endorsed by the publisher.
